# The Clinical Effects of Intravascular Ultrasound-Guided Percutaneous Coronary Intervention in Patients with Chronic Total Occlusion: A Meta-Analysis

**DOI:** 10.1155/2022/4170060

**Published:** 2022-03-17

**Authors:** Zhaoshuang Zhong, Long Zhao, Kaiming Chen, Shuyue Xia

**Affiliations:** ^1^Department of Respiratory, Central Hospital, Shenyang Medical College, Shenyang, China; ^2^Department of Cardiovascular Disease, Central Hospital, Shenyang Medical College, Shenyang, China

## Abstract

**Background:**

The clinical effects of intravascular ultrasound (IVUS)-guided percutaneous coronary intervention (PCI) in patients with chronic total occlusion (CTO) lesions remain unclear.

**Methods:**

We identified all full-text published studies that compared the effects of IVUS-guided CTO-PCI with angiography-guided CTO-PCI by searching electric databases including PubMed, Embase, Cochrane Library, and ISI Web of Science from the establishment to Nov 2021. There was no language limitation. The endpoints included the incidence of major adverse cardiac events (MACE), cardiac death, all-cause death, myocardial infarction (MI), and target vessel revascularization (TVR).

**Results:**

Five studies involving a total of 2320 patients were included in this meta-analysis. Compared to the angiography-guided group, IVUS-guided PCI showed no significant reduction in the incidence of MACE (*I*^2^ = 27.4%, *P* = 0.239; RR 0.929, 95% CI 0.765 to 1.128, *P* = 0.457), cardiac death (*I*^2^ = 0.0%, *P* = 0.459; RR 0.574, 95% CI 0.299 to 1.103, *P* = 0.096), all-cause death (*I*^2^ = 0.0%, *P* = 0.964; RR 0.677, 95% CI 0.395 to 1.163, *P* = 0.158), MI (*I*^2^ = 46.7%, *P* = 0.131; RR0.836, 95% CI 0.508 to 1.377, *P* = 0.482), and TVR (*I*^2^ = 21.2%, *P* = 0.279; RR 0.929, 95% CI 0.679 to 1.272, *P* = 0.648).

**Conclusions:**

IVUS-guided PCI demonstrated no significant benefit on MACE, cardiac death, all-cause death, MI, and TVR in patients with CTO lesions. However, given the study's limitations, additional high-quality RCTs are needed.

## 1. Introduction

Percutaneous recanalization of chronic total occlusion (CTO) remains one of the most challenging issues in interventional cardiology, even in the era of drug-eluting stents (DESs) [1, 2]. As a wildly used technology, intravascular ultrasound (IVUS) guidance can provide a more accurate evaluation of the lesion's morphological features and procedural information and has been proven to be associated with beneficial effects in percutaneous coronary intervention (PCI) therapy [3–5]. However, though IVUS guidance was effective in complex coronary lesions [6, 7], its superiority in the interventional treatment of CTO lesions was not established. Thus, we performed this meta-analysis to compare the clinical effects of IVUS-guided PCI with conventional angiography-guided intervention in patients with CTO lesions.

## 2. Methods

This meta-analysis was conducted according to the Preferred Reporting Items for Systematic Reviews and Meta-Analyses (PRISMA) statement [8].

### 2.1. Literature Search

We conducted a comprehensive literature search of all published articles without time and language limitations through Nov 2021, using the following major electronic databases: PubMed, Embase, Cochrane Library, and ISI Web of Science. Search terms included the keywords of “chronic total occlusion” and “intravascular ultrasound.”

### 2.2. Study Selection

Studies were included if they met the following criteria: (1) randomized controlled trial (RCT) or cohort study, (2) IVUS-guided PCI was performed and compared with conventional angiography-guided intervention for patients with CTO lesions (defined as Thrombolysis In Myocardial Infarction flow grade 0 and occlusion duration > three months [9, 10]), and (3) included at least one of the following clinical outcomes: major adverse cardiac events (MACE, as defined by the authors of the enrolled trials), cardiac death, all-cause death, myocardial infarction (MI), and target vessel revascularization (TVR). Letters, comments, and meeting abstracts were excluded from this meta-analysis.

### 2.3. Data Abstraction and Quality Assessment

Two reviewers (Z.S.Z. and L.Z.) used a predesigned form to extract data from the enrolled studies independently. The extracted data included author, publication year, age, sample size, type of stent, target vessel, intervention strategy, length of follow-up, and the incidence of MACE, cardiac death, all-cause death, MI, and TVR. The methodological qualities of the included trials were evaluated according to the Modified Jadad scale scoring by randomization, double blinding, withdrawals and dropouts, and allocation concealment [11]. In case of discrepancies, a consensus was made by the referral to the senior author (S.Y.X.).

### 2.4. Statistical Analysis

All data were analyzed using STATA version 12.0 (Stata Corp, College Station, TX, USA) with the metan function. We calculated the pooled risk ratios (RRs) with 95% confidence intervals (CI) for dichotomous outcomes and used the *I*^2^ test to assess the heterogeneity among the studies. A fixed-effects (FE) model would be applied if *I*^2^ ≤ 50%. In case of significant heterogeneity (50%<*I*^2^ ≤ 75%), the sensitivity analysis or the subgroup analysis would be considered. A random-effects (RE) model would be applied if heterogeneity remained significant. The data would be treated as unsuitable for pooling in the case of *I*^2^>75% [12]. The publication bias was assessed using funnel plots with Begg's test [13]. A two-sided *P*-value <0.05 indicated a statistical significance.

## 3. Results

### 3.1. Selected Studies and Baseline Characteristics

**Figure 1:**
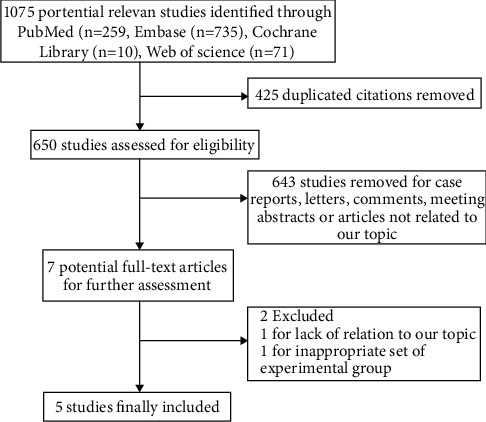
Flow chart of study selection.

Our Literature search strategy led to an initial identification of 1075 records, of which 425 duplicate records were removed. After the title and abstract screening, a further 643 records were excluded as case reports, letters, comments, meeting abstracts, or articles not related to our topic. After full-text browsing, another two records were removed for lack of relation to our topic or inappropriate set of an experimental group [14, 15]. Finally, five studies involving 2320 cases were included in the meta-analysis [16–20]. The flow of the literature selection process is illustrated in Figure 1. The baseline characteristics of selected studies are provided in Table 1, and the angiographic and procedural characteristics of enrolled studies are presented in Table 2.

### 3.2. Quality Assessment and Publication Bias

**Figure 2:**
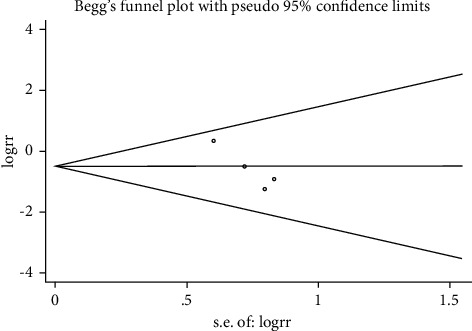
Funnel plot for the events of cardiac death.

We used the Modified Jadad scale to evaluate the quality of the included literature, and the scores are summarized in Table 3. The publication bias risk was assessed using a funnel plot based on the outcome of cardiac death (Figure 2), and no publication bias was found (Begg's test, *P* = 0.221).

### 3.3. Meta-Analysis Results

#### 3.3.1. MACE

**Figure 3:**
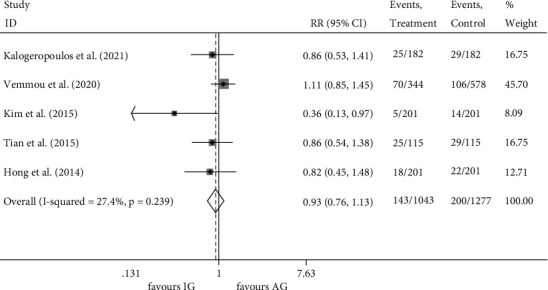
RR of the events of MACE. MACE, major adverse cardiac events; RR, relative risk.

All studies [16–20] reported the incidence of MACE, and no heterogeneity was found among the studies (*I*^2^ = 27.4%, *P* = 0.239). MACE rate was 13.71% (143/1043) in the IVUS-guided group and 15.66% (200/1277) in the angiography-guided group. The results showed no significant reduction of MACE in the IVUS-guided group (RR 0.929, 95% CI 0.765 to 1.128, *P* = 0.457) (Figure 3).

#### 3.3.2. Cardiac Death

**Figure 4:**
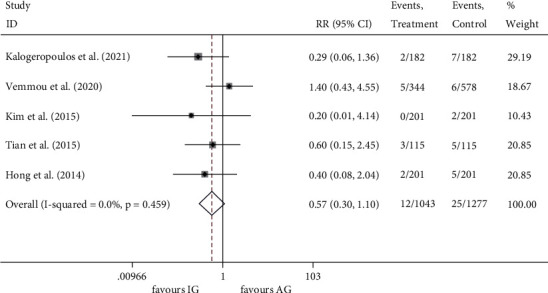
RR of the events of cardiac death. RR, relative risk.

All five studies [16–20] reported the incidence of cardiac death. The events rate was 1.15% (12/1043) in the IVUS-guided group and 1.96% (25/1277) in the angiography-guided group. There was a trend towards a decrease of cardiac death (RR 0.574, 95% CI 0.299 to 1.103, *P* = 0.096) in the IVUS-guided group, but it did not reach statistical significance (Figure 4). The FE model was applied since there was no heterogeneity across the studies (*I*^2^ = 0.0%, *P* = 0.459).

#### 3.3.3. All-Cause Death and MI

**Figure 5:**
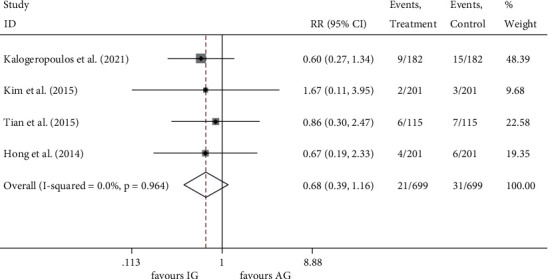
RR of the events of all-cause death. RR, relative risk.

**Figure 6:**
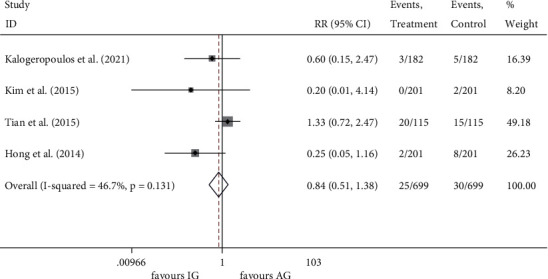
RR of the events of MI. MI, myocardial infarction; RR, relative risk.

Four enrolled studies [16, 18–20] reported all-cause death (*I*^2^ = 0.0%, *P* = 0.964) and MI (*I*^2^ = 46.7%, *P* = 0.131) incidence with no significant heterogeneity among the studies. Compared to the angiography-guided group, IVUS-guided PCI showed no significant reduction in the incidence of all-cause death (RR 0.677, 95% CI 0.395 to 1.163, *P* = 0.158) (Figure 5) and MI (RR0.836, 95% CI 0.508 to 1.377, *P* = 0.482) (Figure 6).

#### 3.3.4. TVR

**Figure 7:**
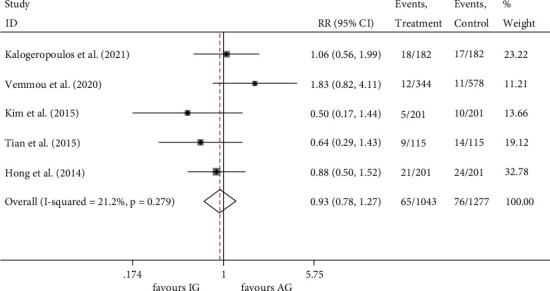
RR of the events of TVR. TVR, target vessel revascularization; RR, relative risk.

The incidence of TVR was investigated in all five studies [16–20]. The events rate was 6.23% (65/1043) in the IVUS-guided group and 5.95% (76/1277) in the angiography-guided group, indicating no significant difference between the two groups (RR 0.929, 95% CI 0.679 to 1.272, *P* = 0.648) (Figure 7). The FE model was used (*I*^2^ = 21.2%, *P* = 0.279).

## 4. Discussion

Several previous studies have reported the advantages of IVUS-guided PCI on coronary vascular disease [21], and meta-analyses further proved its beneficial effects on clinical outcomes in the DES era [22, 23]. A similar relation was expected between CTO lesions and IVUS-guided intervention [24, 25], which led to a broad application of IVUS in the PCI procedure of CTO lesions. However, there are still controversies on the clinical outcomes of IVUS-guided PCI in these patients. On this basis, we conducted this meta-analysis to evaluate the clinical effects of IVUS-guided with angiography-guided PCI in patients with CTO lesions.

Our study, including five studies and a total of 2320 cases, demonstrated that IVUS-guided PCI could not improve the incidence of MACE, cardiac death, all-cause death, MI, and TVR in patients with CTO. In previous studies, IVUS-guided PCI was associated with decreased stent thrombosis [26, 27], which might result from a reduction of procedure-related complications, such as stent underexpansion, malapposition, and incomplete lesion coverage [28–31]. Nevertheless, in the present meta-analysis, the IVUS-guided CTO-PCI showed no significant benefit on clinical outcome indicators compared to angiography-guided treatment. The potential explanation still needs further investigation.

As we all know, IVUS can provide more detailed information on the lesion morphology than angiography-guided intervention, such as reference lumen dimension and lesion length. During the PCI procedure, IVUS is helpful to identify the occlusion point, facilitate the passage of wire in cases with a nontapered stump and side-branches nearby the occlusion site [32], ensure a wire in the true lumen and guide the sub-intimal wire into the true lumen after lesion crossing [33], or apply a reverse controlled antegrade and retrograde tracking (CART) technique [34]. However, it should be noted that one of the main characteristics of CTO lesions is calcification. As reported, calcium can be detected in up to 96% of CTO lesions, which may affect the effectiveness of IVUS [35]. The mechanisms leading to coronary calcification may involve the death of inflammatory cells, the release of matrix vesicles, the differentiation of pericytes or vascular smooth muscle cells (VSMCs), and the impact of genetic such as *β*2-AR signaling [36]. Calcified CTO was associated with longer procedure and fluoroscopy time, lower technical and procedural success rates, and higher incidence of major adverse cardiac events [37]. Given the influence of calcification in CTO lesions, some other techniques such as coronary computed tomography angiography (CCTA) [38] and sub-intimal plaque modification (SPM) [39] may be helpful to predict or improve the success rate of attempted PCI.

Some limitations of this study should not be ignored. Firstly, only five trials were enrolled in the present meta-analysis, including three retrospective studies. Though no statistical heterogeneities were observed among the studies, the analysis's power might be restricted due to the limited study number and population size. Secondly, we enrolled two RCTs that did not report the blind method and allocation concealment in detail, leading to potential bias in the present study. Thirdly, this meta-analysis contained trials regardless of patients' condition, the type of stents implanted, the guidance criteria of IVUS procedure, the duration of follow-up, and the occlusion location, which may also influence the outcomes. For these reasons, the study results should be interpreted with care, and more high-quality RCTs are needed.

## 5. Conclusion

IVUS-guided PCI demonstrated no significant benefit on MACE, cardiac death, all-cause death, MI, and TVR in patients with CTO lesions. Given the study's limitations, the findings should be interpreted with caution, and additional high-quality RCTs are needed.

## Figures and Tables

**Table 1:** Characteristics of included studies.

Study	Year	Study design	IG/CG	Sample size, *n*	Age, *y*	Males, %	Hypertension, %	Diabetes,%	Dyslipidemia, %	Smokers, %	Follow-up
Kalogeropoulos et al. [16]	2021	Observational	IG	182	66.5 (57–72.3)	83.5	69.8	22.5	83.5	25.8	49 months (33.0–67.0)
			CG	182	66.0 (58.0–72.0)	84.6	70.3	22.0	80.8	24.2	

Vemmou et al. [17]	2020	Observational	IG	344	64.9 ± 9.8	82.2	92.0	50.4	93.5	24.7	141 days (30–365)
			CG	578	64.8 ± 9.7	82.8	89.9	51.6	94.8	20.5	

Kim et al. [18]	2015	RCT	IG	201	61.0 ± 11.1	80.6	62.7	34.8	NR	35.3	12 months
			CG	201	61.4 ± 10.1	80.6	63.7	33.8		34.3	

Tian et al. [19]	2015	RCT	IG	115	67 ± 10	88.7	74.8	29.6	21.9	39.1	24 months
			CG	115	66 ± 11	80	70.4	27	27.8	39.1	

Hong et al. [20]	2014	Observational	IG	201	62 ± 11	77	58	30	42	29	12 months
			CG	201	62 ± 12	77	60	31	43	31	

IG, intravascular ultrasound-guided group; AG, angiography-guided group; RCT, randomized controlled trial; NR, not reported. Values are presented as mean ± SD or interquartile range.

**Table 2:** Angiographic and procedural characteristics.

Study	IG/AG	Second-generation DES, %	CTO vessel, %			Successful strategy, %			Number of stents, *n*	Bilateral injection, %
			LAD	LCX	RCA	AWE	ADR	RA		
Kalogeropoulos et al. [16]	IG	100	25.3	7.1	67.6	60.4	9.3	30.2	2.4(2.0–3.0)	94.0
	AG	100	28.0	9.3	62.6	69.2	9.9	20.9	3.0(2.0–3.0)	90.1

Vemmou et al. [17]	IG	NR	32.8	16.7	49.3	53.5	17.4	28.8	2.0(2.0–3.0)	78.1
	AG		23.5	20.5	54.8	57.1	19.8	21.4	2.0(1.0–3.0)	75.5

Kim et al. [18]	IG	100	41.8	14.4	43.8	93.0		7.0	1.7 ± 0.8	50.2
	AG	100	46.8	15.9	37.3	90.5		9.5	1.6 ± 0.7	45.8

Tian et al. [19]	IG	28	44.3	20.9	34.8	89.6		10.4	1.6 ± 0.9	80.9
	AG	20	36.5	14.8	46.1	80.9		19.1	1.5 ± 0.8	89.6

Hong et al. [20]	IG	100	44	16	40	NR			1.71 ± 0.77	NR
	AG	100	34	25	41				1.41 ± 0.69	

IG, intravascular ultrasound-guided group; AG, angiography-guided group; DES, drug-eluting stent; CTO, chronic total occlusion; LAD, left anterior descending artery; LCX, left circumflex coronary artery; RCA, right coronary artery; AWE, antegrade wire escalation; ADR, antegrade dissection reentry; RA, retrograde approach; NR, not reported. Values are presented as mean ± SD or interquartile range.

**Table 3:** Assessment of methodological quality of included studies [11].

Author	Randomization	Double blinding	Allocation concealment	Withdrawals/dropouts	Scores
Kalogeropoulos et al. [16]	NA	NA	NA	NA	NA
Vemmou et al. [17]	NA	NA	NA	NA	NA
Kim et al. [18]	Yes	Unclear	Unclear	Yes	5
Tian et al. [19]	Yes	Unclear	Unclear	Yes	5
Hong et al. [20]	NA	NA	NA	NA	NA

## Data Availability

The data supporting this meta-analysis are from previously reported studies and datasets, which have been cited. The processed data are available from the corresponding author upon request.
